# Cytoreductive Surgery with Hyperthermic Intraperitoneal Chemotherapy (CRS-HIPEC) of Extraperitoneal Abdominal Disease, is it Appropriate?

**DOI:** 10.1245/s10434-024-16866-6

**Published:** 2025-02-04

**Authors:** Christopher W. Mangieri, Konstantinos I. Votanopoulos, Perry Shen, Edward A. Levine

**Affiliations:** Division of Surgical Oncology, Atrium Wake Forest Baptist Medical Center, Winston-Salem, NC USA

## Abstract

**Introduction:**

Cytoreductive surgery-hyperthermic intraperitoneal chemotherapy (CRS-HIPEC) candidates often have extraperitoneal abdominal disease. Current expert peritoneal surface malignancy (PSM) guidelines recommend that the presence of extraperitoneal disease is a contraindication to CRS-HIPEC.

**Methods:**

We conducted a retrospective review of our institutional appendiceal and colorectal CRS-HIPEC registries. Two study cohorts were constructed: (1) cytoreduction with extraperitoneal abdominal disease, and (2) cytoreductions limited to peritoneal structures alone. The primary study outcome was survival. Subgroup analysis was based on the primary tumor and completeness of cytoreduction.

**Results:**

Overall, 864 CRS-HIPEC cases were evaluated, consisting of 578 appendiceal primaries and 286 colorectal cancers. The extraperitoneal cohort included 101 patients, with 763 patients in the non-extraperitoneal group. The median follow-up time was 13.18 years. The main analysis showed no significant differences in survival times. For overall survival (OS) there was a mean OS time of 5.87 years and a median OS time of 4.43 years for extraperitoneal cytoreductions compared with a mean of 5.90 years and a median of 4.76 years for non-extraperitoneal cytoreductions (*p* = 0.955). Five-year OS rates did not differ at 49.1% versus 49.5% (odds ratio [OR] 1.036, 95% confidence interval [CI] 0.671–1.597, *p* = 0.874). Disease-free survival (DFS) times showed a mean of 4.40 years and a median of 1.93 years for extraperitoneal cases versus a mean of 5.44 years and a median of 3.05 years for non-extraperitoneal cases (*p* = 0.210). Five-year DFS rates also showed no differences (OR 0.894, 95% CI 0.476–1.681, *p* = 0.728). No significant differences in progression-free survival (PFS)Pp times (*p* = 0.061) were reported. Multivariate Cox regression analysis indicated that extraperitoneal CRS was not an independent predictor of OS (hazard ratio [HR] 1.281, 95% CI 0.885–1.854, *p* = 0.190), DFS (HR 1.087, 95% CI 0.694–1.701, *p* = 0.716), or PFS (HR 0.650, 95% CI 0.243–1.738).

**Conclusion:**

We conducted the largest analysis evaluating extraperitoneal cytoreductions, with no significant differences in almost all survival outcomes. We propose that the presence of extraperitoneal abdominal disease is not a contraindication to proceeding with CRS-HIPEC.

**Supplementary Information:**

The online version contains supplementary material available at 10.1245/s10434-024-16866-6.

At the beginning of the 21st century, cytoreductive surgery combined with hyperthermic intraperitoneal chemotherapy (CRS-HIPEC) transitioned from an experimental procedure to a recognized oncologic surgery. Initial landmark trials led by Verwaal et al.^1,2^ and Cashin et al.^3^ legitimized CRS-HIPEC within the academic community. More recently, the PRODIGE7 trial has firmly established CRS as standardized therapy with a significant survival advantage for peritoneal carcinomatosis.^4^ The surgical management of peritoneal surface malignancy (PSM) has become more uniform across institutions with the establishment of several well-recognized PSM expert collectives providing evidence-based standards of care; however, many technical aspects of CRS-HIPEC remain debated, unanswered, and deficiently evaluated.^5–7^

A specific clinical subject matter that has a paucity of research is the appropriateness of CRS-HIPEC for disease outside the peritoneal cavity, specifically cytoreducing extraperitoneal abdominal implants. Literature on CRS-HIPEC for extraperitoneal disease is limited and has primarily been focused on thoracic extension of malignancy or intraparenchymal liver metastases.^8–13^ Thoracic HIPEC for malignant mesothelioma has a defined beneficial role;^14–16^ however, CRS-HIPEC for extraperitoneal tumor spread from the more common etiologies of PSM such as colorectal and appendiceal primaries has conflicting results.^8–13^ In their current guidelines, both the Chicago Consensus Working Group and the Canadian HIPEC Collaborative Group indicate that extraperitoneal disease is a contraindication to proceeding with CRS-HIPEC;^17–19^ however, the definition of extraperitoneal disease in those guidelines is not granular, and nor do the guidelines address the specific subset of abdominal extraperitoneal disease.

This study specifically evaluated the long-term oncologic outcomes of CRS-HIPEC, which included abdominal extraperitoneal cytoreduction (AEC). We suspect that this practice occurs frequently at many PSM centers but has been underreported. To our knowledge, this is the first study to exclusively evaluate AEC with a focus on survival. The objective of this research was to provide meaningful data to help guide the discussion on whether CRS-HIPEC for extraperitoneal abdominal disease has utility or is a futile practice.

## Methods

This analysis was a retrospective review of our prospectively maintained appendiceal and colorectal CRS-HIPEC registries, which was conducted following Institutional Review Board (IRB) approval. The appendiceal registry cases analyzed included low-grade appendiceal mucinous neoplasms (LAMNs) and appendiceal carcinomas, including adenocarcinoma, goblet cell adenocarcinoma, and neuroendocrine tumors. All colorectal cancer cases analyzed were adenocarcinomas. All index cases from the combined databases were extracted, and repeat CRS-HIPEC surgeries were excluded. Two study cohorts were constructed: (1) cytoreductions that only involved intraperitoneal abdominal structures; and (2) cytoreductions that also involved resection of AECs. Thoracic extraperitoneal CRS-HIPEC cases, as well as abdominal cytoreductions that involved the resection of intraparenchymal hepatic metastases, were excluded. Extraperitoneal abdominal structures, including the pancreas, kidney, adrenal gland, and umbilicus, exhibited extension of gross disease into those structures. Inclusion criteria for the extraperitoneal cytoreduction cohort included resection of at least one of those structures. Resection of intraperitoneal structures was also allowed in the extraperitoneal group. All pancreatic resections were distal pancreatectomies, while nephrectomies were either partial or total. All adrenal resections were unilateral complete adrenalectomies. Umbilectomy cases included instances of gross disease clearly present within the extraperitoneal soft tissue of the abdominal wall.

The cohorts were compared based on baseline demographic data, comorbidities, perioperative outcomes, and oncologic parameters. The oncologic parameters included disease burden, measured using the Peritoneal Cancer Index (PCI) score, and completeness of cytoreduction. At our institution, the R1, R2a, R2b, and R2c cytoreductive scheme is used for completeness of cytoreduction classification; R1 indicates no gross residual disease, R2a indicates gross residual disease <2.5 mm, R2b indicates gross residual disease between 2.5 and 25 mm, and R2c indicates gross residual disease >25 mm. Complete cytoreductions were defined as R1 and R2a, while R2b and R2c were classified as incomplete cytoreductions. Previously published data have shown that R1 and R2a cytoreductions have similar clinical outcomes, specifically in regard to survival.^20,21^

The institutional protocol for all appendiceal and colorectal HIPEC cases involves perfusion with either oxaliplatin or mitomycin C. A closed technique is performed with a 2-h HIPEC run time, with the chemotherapy agents added once the outflow temperature exceeds 39 °C. The target outflow temperature during the HIPEC perfusion is 40 °C, with a maximum tolerated inflow temperature of 42.5 °C. For oxaliplatin cases, a single dose of 200 mg/m^2^ is utilized, and for mitomycin C perfusions, an initial dose of 30 mg is administered, followed by an additional 10 mg dose at the 1-h mark. All analyzed cases followed this protocol. The standard institutional practice is to not perfuse with HIPEC for most R2b cytoreductions and all R2c cytoreductions (incomplete cytoreductions) due to the proposed ineffectiveness of chemotherapy penetration for the remaining gross disease and the current expert consensus recommendations.^22^ However, HIPEC is an effective palliative agent for malignant ascites, thus a number of our patients with malignant ascites still receive HIPEC with incomplete cytoreductions.^23,24^ Ultimately, it is at the discretion of each individual HIPEC surgeon to decide whether or not to proceed with perfusion in cases of incomplete cytoreductions.

The primary study endpoints were survival outcomes, measured by overall survival (OS), disease-free survival (DFS), and progression-free survival (PFS), and reported as mean and median survival times. All survival analyses excluded cases with 90-day mortalities. When evaluating DFS outcomes cases, those who underwent an incomplete cytoreduction (R2b, R2c) were excluded. For the PFS analysis only, incomplete cytoreductions were included. For both DFS and PFS, disease progression was determined by surveillance imaging or gross clinical evidence of progressive disease. Additional survival investigation included actuarial 5-year survival rates. Subgroup analyses were also performed based on primary tumor etiology and completeness of cytoreduction. Multivariate analysis included factors that have been previously established to significantly affect survival outcomes following CRS-HIPEC.

### Statistical Analysis

Categorical variables were evaluated using Pearson’s Chi-square testing.  Quantitative variables were evaluated using Fishers’s exact testing.  Both the Pearson’s and Fisher’s evaluations were univariate testing with two-tailed reporting of *p*-values.  Multivariate analysis was performed using Cox regression to report hazard ratios (HRs). Kaplan–Meier survival curves were also calculated, and projected survival times were compared using log-rank analysis. Odds ratios (ORs) associated with 5-year survival rates are derived from univariate evaluations. All *p*-values <0.05 were considered significant. All descriptive statistics were processed using the IBM Statistical Package for Social Sciences (SPSS) version 28.0.0.0 analytical software (IBM Corporation, Armonk, NY, USA).

## Results

A total of 864 index CRS-HIPEC cases met the study inclusion criteria, of which 578 were appendiceal primaries and 286 were colorectal cancers. Within the appendiceal primaries, 475 cases had a mucinous component, of which 321 were LAMNs. There were 249 appendiceal carcinoma cases, with 154 cases being mucinous carcinomas and the remaining 95 being non-mucinous carcinoma tumors. The AEC cohort included 101 patients, while the non-extraperitoneal group consisted of 763 patients. Within the AEC cohort, there were 54 pancreatectomies, 45 umbilectomies, 7 nephrectomies, 6 adrenalectomies, and 11 cases involving resection of multiple extraperitoneal structures. All AECs also involved resection of intraperitoneal structures. The median follow-up time for all patients was 13.18 years, with median times of 11.61 and 13.26 years for the extraperitoneal and non-extraperitoneal groups. Characteristics of the two cohorts are provided in Table 1. The two study cohorts had statistically equivalent baseline characteristics, with both groups consisting of Caucasians in their mid-50s who had mild medical comorbidities. There were no differences between the groups in terms of cardiopulmonary disease, diabetic status, and smoking history; however, several significant differences in the cohorts were present. The extraperitoneal group included more males who exhibited decreased functional status, with a greater proportion of appendiceal primaries and a greater burden of peritoneal disease burden. Additionally, this group achieved fewer complete cytoreductions and had a higher rate of chemotherapy utilization. There was no statistical difference in postoperative complications or other significant baseline oncologic characteristics. Regarding chemotherapy treatment, 95% of colorectal cases received chemotherapy; 91% received neoadjuvant therapy, 42% received adjuvant therapy, and 37% received both neoadjuvant and adjuvant therapy. For appendiceal cases, 38% received chemotherapy; 31% received neoadjuvant therapy, 12% received adjuvant therapy, and 6% received both neoadjuvant and adjuvant therapy. For both appendiceal and colorectal cases, the overwhelming majority of patients received multiagent 5-fluorouracil-based regimens (FOLFOX, FOLFIRI). On average, colorectal patients received 6 months of therapy, while the duration of therapy for appendiceal cases varied between 3 and 6 months.

**Table 1:** Cohort demographics

	Extraperitoneal CRS [*n* = 101 patients]	Non-extraperitoneal CRS [*n* = 763 patients]	*p*-Value
Age, years	55.27 (±12.64)	53.04 (±12.01)	0.087
Sex^a^			
Male Female	58.0% 42.0%	44.7% 55.3%	0.012
Race			
Caucasian Non-Caucasian	83.2% 16.8%	86.4% 13.6%	0.384
BMI	28.48 (+/-6.12)	28.08 (+/-6.36)	0.639
ASA Class	ASA-2, 78.9% ASA-3, 21.2% ASA-4, 0.0%	ASA-2, 82.3% ASA-3, 15.4% ASA-4,2.3%	0.233
ECOG status^a^			
ECOG-0 ECOG-1 ECOG-2 ECOG-3	38.9% 41.1% 14.7% 5.3%	56.1% 34.8% 7.7% 1.2%	0.003
Cardiovascular disease	13.6%	8.2%	0.136
Pulmonary disease	1.5%	3.8%	0.343
Diabetic	9.1%	8.5%	0.871
Smoker	26.2%	32.4%	0.306
Major complications (Clavien–Dindo Grade III or higher)	25.7%	17.6%	0.670
PCI score^a^	17.82 (±9.50)	13.06 (±8.73)	<0.001
Cytoreduction score^a^			
R1 R2a R2b R2c	38.6% 28.7% 26.7% 5.9%	52.2% 28.6% 11.1% 6.7%	0.001
Complete cytoreduction^a^	67.3%	81.9%	<0.001
Primary tumor^a^			
Appendiceal Colorectal	82.2% 17.8%	64.9% 35.1%	<0.001
Histologic disease grade			
Low High	62.8% 37.2%	58.2% 41.8%	0.419
Lymph node-positive disease	33.3%	44.5%	0.080
Chemotherpy utilization^a^	43.6%	58.6%	0.005

^a^Statistically significant

*CRS* cytoreductive surgery, *BMI* body mass index, *ECOG* Eastern Cooperative Oncology Group, *PCI* Peritoneal Cancer Index

**Fig. 1:**
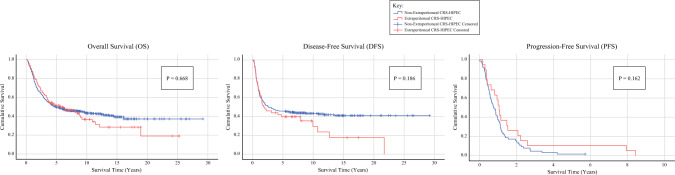
Kaplan–Meier survival curves. *CRS* cytoreductive surgery, *HIPEC* hyperthermic intraperitoneal chemotherapy

For the entire study population, survival outcomes showed no significant differences in survival times between the cohorts across all measures, including OS, DFS, and PFS. The survival times and 5-year survival rates are demonstrated in Tables 2 and 3, respectively. OS times were nearly identical, with a mean of 5.87 years and a median of 4.43 years for AECs compared with a mean of 5.90 years and a median of 4.76 years for non-extraperitoneal cytoreductions (*p* = 0.955). Similarly, the 5-year OS rates for both groups were comparable at 49.1% and 49.5% for extraperitoneal and non-extraperitoneal cases, respectively (OR 1.036, 95% CI 0.671–1.597, *p* = 0.874). Overall, 684 cases with complete cytoreductions were available for DFS analysis, of which 68 were AECs and 616 were non-extraperitoneal cytoreductions. For PFS evaluation, a total of 169 incomplete cytoreduction cases were present; 33 extraperitoneal cases and 136 non-extraperitoneal cases. Furthermore, DFS times and 5-year DFS rates did not statistically differ between the cohorts; however, for PFS, the AEC cases exhibited longer survival times that approached significance (*p* = 0.061), and 5-year PFS rates were statistically improved at 13.6% versus 2.2% (OR 6.87, 95% CI 1.073–43.978, *p* = 0.021). Kaplan–Meier survival curve projections are illustrated in Fig. 1. The survival curves mirrored the OS, DFS, and PFS time results, showing no significant differences in survival projections.

**Table 2:** Survival times

	Mean survival (years)	Median survival (years)	*p*-Value
*Overall survival*			
No extraperitoneal CRS	5.90 (±5.19)	4.76	0.955
Extraperitoneal CRS	5.87 (±5.30)	4.43	
*Disease-free survival*			
No extraperitoneal CRS	5.44 (±5.59)	3.05	0.210
Extraperitoneal CRS	4.40 (±4.86)	1.93	
*Progression-free survival*			
No extraperitoneal CRS	1.13 (±1.47)	0.73	0.061
Extraperitoneal CRS	2.40 (±3.10)	1.12	

*CRS* cytoreductive surgery

**Table 3:** Actuarial 5-year survival rates

	No extraperitoneal CRS	Extraperitoneal CRS	OR	95% CI	*p*-Value
Actuarial 5-year OS rate	49.5%	49.1%	1.036	0.671–1.597	0.874
Actuarial 5-year DFS rate	47.0%	44.2%	0.894	0.476–1.681	0.728
Actuarial 5-year PFS rate	2.2%	13.6%	6.87	1.073–43.978	0.021

*OS* overall survival, *DFS* disease-free survival, *PFS* progression-free survival, *CRS* cytoreductive surgery, *OR* odds ratio, *CI* confidence interval

The results of the multivariate Cox regression analysis for survival are provided in Table 4. Performing an AEC was not found to be significant with any survival metrics evaluated. Primary tumor type was the most significant negative independent factor for OS, with colorectal cancers having a median OS of 1.94 years compared with 6.71 years for appendiceal tumors (HR 3.257, 95% CI 2.367–4.482, *p* < 0.001). High-grade histopathology, lymph node positivity, higher PCI score, and male sex were also independent risk factors for decreased OS. Achieving a complete cytoreduction was the only variable associated with significantly improved OS (HR 0.494, 95% CI 0.326–0.747, *p* < 0.001). Regarding DFS, colorectal cancer most significantly decreased survival (HR 8.93, 95% CI 5.909–13.496, *p* < 0.001) followed by nodal disease and higher PCI scores. For PFS, nodal positivity was the only significant independent factor associated with survival. A multivariate analysis was conducted to assess factors associated with achieving a complete cytoreduction, including primary tumor, AEC, PCI score ≥20, histopathologic grade, and lymph node status. In that analysis, only a PCI score ≥20 was significantly associated with completeness of cytoreduction, with a high PCI score associated with a decreased ability to achieve a complete cytoreduction (HR 0.071, 95% CI 0.037–0.137, *p* < 0.001). The performance of an AEC was not associated with completeness of cytoreduction (HR 0.838, 95% CI 0.384–1.828, *p* = 0.657). The complete results of the multivariate analysis for completeness of cytoreduction are provided in electronic supplementary material (ESM) Table 1.

**Table 4:** Cox regression multivariate analysis

	HR	95% CI	*p*-Value
*Overall survival*			
Age	1.005	0.994–1.016	0.395
Race	1.177	0.813–1.703	0.388
Sex^a^	1.323	1.034–1.694	0.026
Histopathologic grade^a^	1.381	1.058–1.803	0.018
Lymph node positivity^a^	2.317	1.717–3.126	<0.001
Extraperitoneal cytoreduction	1.281	0.885–1.854	0.190
Primary tumor^a^	3.257	2.367–4.482	<0.001
Complete cytoreduction^a^	0.494	0.326–0.747	<0.001
PCI score^a^	1.019	1.002–1.037	0.033
*Disease-free survival*			
Age	1.002	0.989–1.014	0.804
Race	0.837	0.533–1.315	0.441
Sex	1.049	0.780–1.410	0.753
Histopathologic grade	1.360	0.968–1.910	0.077
Lymph node positivity^a^	1.631	1.122–2.371	0.010
Extraperitoneal cytoreduction	1.087	0.694–1.701	0.716
Primary tumor^a^	8.930	5.909–13.496	<0.001
PCI score^a^	1.036	1.015–1.058	<0.001
*Progression-free survival*			
Age	0.996	0.969–1.024	0.796
Race	0.384	0.127–1.156	0.089
Sex	0.977	0.379–2.516	0.961
Histopathologic grade	1.550	0.541–4.436	0.414
Lymph node positivity^a^	0.292	0.091–0.940	0.039
Extraperitoneal cytoreduction	0.650	0.243–1.738	0.390
Primary tumor	1.536	0.413–5.711	0.522
PCI score	1.004	0.953–1.059	0.873

^a^ Statistically significant

*HR* hazard ratio, *CI* confidence interval, *PCI* Peritoneal Cancer Index

Lastly, subgroup analysis based on primary tumor type and completeness of cytoreduction was performed. The survival time results for all subgroup analyses are provided in Table 5. For the 578 appendiceal tumor cases, 83 cases included an AEC, with the remaining 495 cases only involving cytoreduction of intraperitoneal structures. Within the appendiceal subgroup, there were 440 cases with complete cytoreductions, which allowed for the evaluation of DFS. Among these cases, 51 and 389 cases were classified as AECs and non-extraperitoneal cytoreductions, respectively. Overall, 128 incomplete appendiceal cytoreductions were analyzed for PFS, involving 32 and 96 extraperitoneal and non-extraperitoneal cases, respectively. Additionally, there were 10 appendiceal cases, all non-extraperitoneal cytoreductions, for which completeness of cytoreduction was not available, therefore only 568 total cases were available for completeness of cytoreduction subgroup analysis. There was no statistically significant difference in OS times between AECs and non-extraperitoneal cytoreductions (*p* = 0.169), nor any difference in 5-year OS rates, at 56.6% and 62.6% (OR 0.778, 95% CI 0.477–1.268, *p* = 0.314); however, there was a significant difference in DFS times, with AECs having inferior survival (*p* = 0.040). The actuarial 5-year DFS rates for appendiceal cases were not statistically significant, at 58.1% and 66.8% for extraperitoneal and non-extraperitoneal cases, respectively (OR 0.689, 95% CI 0.324–1.464, *p* = 0.327). Evaluation of PFS revealed no significant differences in survival times (*p* = 0.160) or survival rates (OR 4.26, 95% CI 0.661–27.495, *p* = 0.133) for appendiceal cases. For appendiceal primaries, more second-order subgroup analyses were performed based on the histopathologic subtype of LAMNs, mucinous carcinomas, and non-mucinous carcinomas. In the LAMN subgroup analysis, there were no differences in any survival outcomes, with OS at 9.0 and 8.2 years (*p* = 0.310), DFS at 8.4 and 7.6 years (*p* = 0.511), and PFS at 1.7 and 2.6 years (*p* = 0.246) for non-extraperitoneal and extraperitoneal cases, respectively. For mucinous carcinomas, there were also no differences in survival, with OS at 5.2 and 4.1 years (*p* = 0.343), DFS at 5.4 and 3.2 years (*p* = 0.341), and PFS at 1.0 and 1.6 years (*p* = 0.522) for non-extraperitoneal and extraperitoneal cases, respectively. Lastly, for non-mucinous carcinomas, there was inferior survival associated with AECs, for OS at 5.0 and 3.3 years (*p* = 0.030) and DFS at 5.9 and 1.7 years (*p* < 0.001); however AECs demonstrated improved PFS at 3.3 and 0.9 years (*p* = 0.007). The non-mucinous carcinoma subgroup analysis should be interpreted with caution as only 13, 9, and 3 AECs were available for analysis in that subgroup.

**Table 5:** Subgroup survival times

	Mean survival (years)	Median survival (years)	*p*-Value
*Appendiceal OS*			
No extraperitoneal CRS	7.25 (±5.42)	6.90	0.169
Extraperitoneal CRS	6.36 (±5.52)	5.43	
*Appendiceal DFS*			
No extraperitoneal CRS	7.47 (±5.65)	7.26	0.040
Extraperitoneal CRS	5.46 (±5.21)	4.78	
*Appendiceal PFS*			
No extraperitoneal CRS	1.43 (±1.78)	0.98	0.160
Extraperitoneal CRS	2.40 (±3.10)	1.06	
*Colorectal OS*			
No extraperitoneal CRS	3.33 (±3.50)	1.91	0.839
Extraperitoneal CRS	3.51 (±3.18)	2.38	
*Colorectal DFS*			
No extraperitoneal CRS	1.08 (±1.43)	0.67	0.456
Extraperitoneal CRS	1.38 (±1.32)	0.95	
*Colorectal PFS*			
No extraperitoneal CRS	–	–	–
Extraperitoneal CRS	–	–	
*Complete cytoreduction OS*			
No extraperitoneal CRS	6.61 (±5.36)	6.05	0.955
Extraperitoneal CRS	6.57 (±5.64)	5.43	
*Incomplete cytoreduction OS*			
No extraperitoneal CRS	2.83 (±2.97)	1.55	0.044
Extraperitoneal CRS	4.46 (±4.27)	2.95	

*CRS* cytoreductive surgery, *OS* overall survival, *DFS* disease-free survival, *PFS* progression-free survival

There were 286 colorectal cancer cases for that subgroup analysis, with 18 cases involving AECs and 268 cases without an extraperitoneal cytoreduction. The majority of colorectal cases achieved a complete cytoreduction; 244 cases were available for DFS analysis, of which 17 and 227 cases were extraperitoneal and non-extraperitoneal cases, respectively. There were only 41 incomplete colorectal cytoreductions, with only one of these cases being extraperitoneal; therefore, this subset was too small for statistical evaluation of PFS. The colorectal subgroup analysis revealed no significant differences in either OS (*p* = 0.839) or DFS (*p* = 0.456) times. Likewise, the 5-year OS and DFS survival rates were similar at 18.8% and 22.2% (OR 0.810, 95% CI 0.223–2.944, *p* = 1.00), respectively, and 8.3% and 3.0% (OR 2.932, 95% CI 0.301–28.553, *p* = 0.355), respectively, for extraperitoneal versus non-extraperitoneal cases, respectively.

**Fig. 2:**
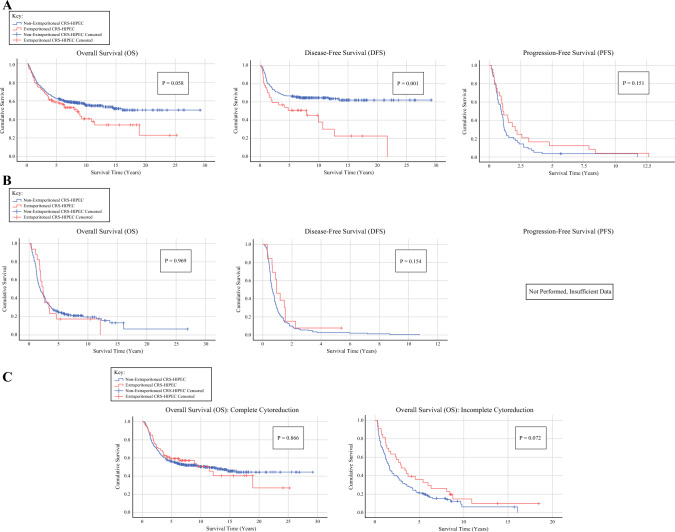
Kaplan–Meier survival curves: **a** appendix subgroup analysis; **b** colorectal subgroup analysis; **c** completeness of cytoreduction subgroup analysis. *CRS* cytoreductive surgery, *HIPEC* hyperthermic intraperitoneal chemotherapy

Finally, for the completeness of cytoreduction subgroup analysis, 684 cases achieved a complete cytoreduction and 169 cases underwent an incomplete cytoreduction. Within the extraperitoneal cohort, there were 68 complete cytoreductions and 33 incomplete cytoreductions. The non-extraperitoneal cases involved 616 and 136 complete and incomplete cytoreductions, respectively. For the complete cytoreduction analysis, there were no significant differences in OS times (*p* = 0.955), and similar 5-year OS rates of 55.3% and 58.3% (OR 1.133, 95% CI 0.661–1.940, *p* = 0.685). The incomplete cytoreduction subgroup evaluation revealed improved OS for the extraperitoneal cohort (*p* = 0.044), however there was no significant difference in 5-year OS rates (OR 2.002, 95% CI 0.864–4.636, *p* = 0.109). No additional subgroup analysis stratified by completeness of cytoreduction was performed as the DFS and PFS examination in the main analysis was already delineated by complete and incomplete cytoreductions, respectively. Kaplan–Meier survival curves for all the subgroups are portrayed in Fig. 2a, b, and c. Those survival projections correlated with the survival time results. A complete description of the subgroup 5-year survival rates can be found in ESM Table 2.

## Discussion

A salient principle of CRS-HIPEC is that if PSM is limited to the peritoneal cavity, then cytoreducing all gross disease combined with HIPEC for eradication of residual microscopic disease can provide a long-term survival benefit, potentially even curative therapy. The survival benefit of cytoreduction was first demonstrated by Dr. Meigs’ pioneering publications on CRS with ovarian cancer.^25^ That research was further progressed with clinical studies by Dr. Griffiths at the National Cancer Institute evaluating ovarian cancer, and Dr. Long’s research team in Alabama evaluating mucinous neoplasms; both research groups demonstrated improved survival with CRS.^26,27^ Dr. Sugarbaker’s prolific work combining HIPEC with CRS laid the groundwork for the modern practice of PSM.^28–31^ The four published randomized clinical trials to date involving CRS-HIPEC have provided the validating data for PSM surgery.^1–4,32^ All that scientific work centered around the premise that CRS-HIPEC is only effective when disease is limited to the peritoneal cavity.

As collective experience and research with CRS-HIPEC has progressed, the fundamental tenets of PSM surgery have also been further queried. One such expansion of research has been investigating the utility of extraperitoneal cytoreductions with HIPEC. Previous studies evaluating extraperitoneal CRS-HIPEC have largely been limited to thoracic disease or intraparenchymal hepatic metastases.^8–13^ Thus far, objective evidence has only routinely demonstrated efficacy for extraperitoneal CRS-HIPEC for malignant pleural mesothelioma.^14–16^ Both retrospective and prospective studies have shown a survival benefit with thoracic CRS-HIPEC for this select patient population;^15,16^ however, for the more common malignancies associated with peritoneal disease, such as appendiceal and colorectal tumors, the benefits of extraperitoneal CRS-HIPEC have been inconclusive. Thoracic CRS-HIPEC for gastrointestinal primaries has been associated with acceptable surgical morbidity and survival benefit in some analyses, while other investigations have found that thoracic extension of CRS-HIPEC results in significantly increased surgical morbidity and decreased survival.^9–12^ Research regarding AEC for appendiceal and colorectal cancer has been almost exclusively limited to hepatic metastases, with evaluation of hepatectomies performed concurrently with CRS-HIPEC. The literature has also shown mixed results regarding the safety and efficacy of hepatic parenchymal debulking combined with standard cytoreduction at the time of HIPEC.^13,33–36^ Therefore, given the lack of compelling evidence to support the benefits of cytoreduction for extraperitoneal disease during CRS-HIPEC, current expert PSM organizations such as the Chicago Consensus Working Group and the Canadian HIPEC Collaborative Group propose that the presence of extraperitoneal disease is a contraindication to proceeding with CRS-HIPEC.^17–19^ However, as previously mentioned, neither the Chicago Consensus nor the Canadian HIPEC groups specifically address extraperitoneal abdominal disease, therefore no current expert consensus guidelines provide evidence-based recommendations on CRS-HIPEC for extraperitoneal abdominal disease.

This study evaluated CRS-HIPEC for extraperitoneal abdominal disease, an area that has been rarely evaluated in the PSM literature. Nonetheless, it is the anecdotal experience of the authors that AECs are routinely performed at many tertiary HIPEC centers, but this practice is often underreported. Furthermore, we suspect a large majority of HIPEC surgeons would not consider the presence of abdominal extraperitoneal disease as a contraindication to proceeding with CRS-HIPEC. Our institution has previously researched the effect of a distal pancreatectomy performed at the time of CRS-HIPEC in cases where such a resection was required for retroperitoneal extension of disease, both within our individual institutional CRS-HIPEC registry and through collaborative efforts with other HIPEC centers.^37,38^ The primary outcome of those studies focused on the surgical safety and impact on postoperative complications associated with pancreatectomy during CRS-HIPEC. The results indicated that distal pancreatectomy was associated with acceptable morbidity and mortality when included in the cytoreduction process.^37,38^ Limited survival analysis from one of those investigations suggested that if a complete cytoreduction could be achieved with a distal pancreatectomy, then a long-term survival benefit was present, similar to that observed in patients without extraperitoneal abdominal disease who underwent CRS-HIPEC.^37^ Other institutions have also examined the effect of distal pancreatectomies and other AECs on short-term surgical outcomes, without robust oncologic or survival evaluation.^39–41^ After an extensive literature search, we found only one recent retrospective study that focused on long-term survival for CRS-HIPEC involving extraperitoneal disease. That study was from the US HIPEC collaborative evaluating approximately 130 cases of CRS-HIPEC, from a combination of appendiceal and colorectal primaries, involving extraperitoneal cytoreductions compared with over 1000 cases of non-extraperitoneal CRS-HIPEC across 12 academic HIPEC centers.^42^ In the overall analysis, extraperitoneal CRS-HIPEC was associated with significantly inferior OS and DFS; however, on propensity-score matching, there was no significant difference in survival.^42^ Only one-third of extraperitoneal cases in that study involved abdominal extraperitoneal disease exclusive of intraparenchymal hepatic metastases.^42^

To our knowledge, this is the largest analysis evaluating oncologic outcomes and long-term survival for CRS-HIPEC involving AEC. A comparison of 101 extraperitoneal cases with 763 non-extraperitoneal cases revealed no significant differences in OS, DFS, and PFS for the entire study population. Furthermore, inclusion of an AEC was not found to be an independent prognostic factor for any survival outcomes with CRS-HIPEC, or in achieving a complete cytoreduction. The only detrimental survival effect of performing an AEC in this study was limited to DFS for appendiceal primaries. While additional objective research is required to validate these study findings, our results support proceeding with CRS-HIPEC even in the presence of extraperitoneal abdominal disease, assuming that a complete cytoreduction can be achieved despite that disease. If these findings are corroborated by results from other tertiary HIPEC centers, we propose considering removal of the presence of abdominal extraperitoneal disease as a contraindication to CRS-HIPEC, as currently stated in expert consensus guidelines. Furthermore, we propose enhanced work-up to detect abdominal extraperitoneal disease. The extent of disease in PSM is often underestimated on preoperative imaging, even with current high-resolution cross-sectional imaging.^43,44^ Magnetic resonance imaging (MRI) has shown greater accuracy over computed tomography (CT) when evaluating extraperitoneal disease and enhancing surgical planning for CRS-HIPEC.^44^ MRI should be considered in the preoperative work-up of potential CRS-HIPEC patients with suspected extraperitoneal disease.

Limitations are present within this analysis. As this was a retrospective investigation, the study findings should be interpreted as indicating no significant association between performing an AEC and survival outcomes, as opposed to directly stating that performing an AEC definitively has no impact on survival. Additionally, there was significant heterogenicity between the study cohorts. Notably, the AEC group had a significantly heavier disease burden and a decreased complete cytoreduction rate. This inferior complete cytoreduction rate is typically associated with inferior survival. However, the AECs also had a higher proportion of appendiceal cases, which have improved survival outcomes compared with colorectal cancer cases. While there were significant differences in the baseline complete cytoreduction rates and primary tumor etiologies between the study groups during subgroup analysis, when stratified by primary tumor and completeness of cytoreduction, only a significantly decreased DFS was found with AEC. Despite the inherent limitations of this study, we still propose that the findings hold clinical merit. Further validation with either multi-institutional reviews or prospective analyses is warranted to confirm these results.

## Conclusion

The presence of extraperitoneal abdominal disease is not an infrequent finding for potential CRS-HIPEC candidates with appendiceal and colorectal cancer. Due to limited research on the questionable utility of CRS-HIPEC in this specific patient subset, leading PSM expert organizations currently consider the presence of extraperitoneal disease as a contraindication to proceeding with surgical intervention, without specifically addressing abdominal extraperitoneal disease. This is the largest analysis to date exclusively evaluating the effect of AEC, a practice that is likely common at many HIPEC centers. The study results found no significant difference in any survival outcomes for patients undergoing CRS-HIPEC with abdominal extraperitoneal disease. Furthermore, an AEC was not found to be a significant factor regarding survival following CRS-HIPEC on multivariate analysis. While our study results require additional academic validation, we propose that the presence of extraperitoneal abdominal disease is not a contraindication to CRS-HIPEC. Achieving an optimal cytoreduction with extraperitoneal abdominal disease appears to have equivocal survival benefit to cases with disease limited to peritoneal structures alone.

## Supplementary Information

Below is the link to the electronic supplementary material.

Supplementary file1 (DOCX 66 KB)
