# Supplementary material for: Cytoreductive Surgery with Hyperthermic Intraperitoneal Chemotherapy (CRS-HIPEC) of Extraperitoneal Abdominal Disease, is it Appropriate?
Source: Ann Surg Oncol. 2025 Feb 4;32(4):2893–902. doi: 10.1245/s10434-024-16866-6 (PMC11882691; doi:10.1245/s10434-024-16866-6)
Supplement: Supplementary file 1 — Supplementary file1 (DOCX 66 KB) [file 10434_2024_16866_MOESM1_ESM.docx]

**Supplemental Table:**

Table 1: Cox-Regression Multivariate Analysis Completeness of Cytoreduction (achieving a complete cytoreduction)

|  | HR | 95% CI | P Value |
| --- | --- | --- | --- |
| Primary Tumor | 2.160 | 0.927-5.031 | 0.074 |
| Extraperitoneal Cytoreduction | 0.838 | 0.384-1.828 | 0.657 |
| PCI Score (≥20)† | 0.071 | 0.037-0.137 | <0.001 |
| Histopathologic Grade | 0.899 | 0.470-1.718 | 0.746 |
| Lymph Node Positivity | 0.542 | 0.253-1.161 | 0.115 |

†Statistically significant

Table 2: Actuarial 5-Year Survival Rates Subgroups

Actuarial 5-Year OS: Appendix

|  | No Extraperitoneal  CRS | Extraperitoneal  CRS | OR | 95% Confidence  Interval | P  value |
| --- | --- | --- | --- | --- | --- |
| 5-Year Overall  Survival Rate (%) | 62.6% | 56.6% | 0.778 | 0.477-1.268 | 0.314 |

Actuarial 5-Year DFS: Appendix

|  | No Extraperitoneal  CRS | Extraperitoneal  CRS | OR | 95% Confidence  Interval | P  value |
| --- | --- | --- | --- | --- | --- |
| 5-Year Disease-Free  Survival Rate (%) | 66.8% | 58.1% | 0.689 | 0.324-1.463 | 0.327 |

Actuarial 5-Year PFS: Appendix

|  | No Extraperitoneal  CRS | Extraperitoneal  CRS | OR | 95% Confidence  Interval | P  value |
| --- | --- | --- | --- | --- | --- |
| 5-Year Progression-Free  Survival Rate (%) | 3.6% | 13.6% | 4.26 | 0.661-27.495 | 0.133 |

Actuarial 5-Year OS: Colorectal

|  | No Extraperitoneal  CRS | Extraperitoneal  CRS | OR | 95% Confidence  Interval | P  value |
| --- | --- | --- | --- | --- | --- |
| 5-Year Overall  Survival Rate (%) | 22.2% | 18.8% | 0.810 | 0.223-2.944 | 1.00 |

Actuarial 5-Year DFS: Colorectal

|  | No Extraperitoneal  CRS | Extraperitoneal  CRS | OR | 95% Confidence  Interval | P  value |
| --- | --- | --- | --- | --- | --- |
| 5-Year Disease-Free  Survival Rate (%) | 3.0% | 8.3% | 2.932 | 0.301-28.553 | 0.355 |

Actuarial 5-Year PFS: Colorectal

Not performed insufficient data

Actuarial 5-Year OS: Complete Cytoreduction

|  | No Extraperitoneal  CRS | Extraperitoneal  CRS | OR | 95% Confidence  Interval | P  value |
| --- | --- | --- | --- | --- | --- |
| 5-Year Overall  Survival Rate (%) | 55.3% | 58.3% | 1.133 | 0.661-1.940 | 0.685 |
